# Effect of TiO_2_ as an additive on the sintering performance of Sm-doped CeO_2_-based electrolyte for solid oxide fuel cells

**DOI:** 10.3389/fchem.2022.1034993

**Published:** 2022-10-06

**Authors:** Xuzhuo Sun, Shuailei Deng, Yunyun Xia, Bo Li, Ye Tian, Jing Chen

**Affiliations:** ^1^ School of Chemistry and Chemical Engineering, Henan University of Technology, Zhengzhou, China; ^2^ School of Mechanical and Electrical Engineering, Henan University of Technology, Zhengzhou, China

**Keywords:** solid oxide fuel cell, Sm-doped CeO_2_, grain boundary, TPB, electrolyte

## Abstract

In this work, TiO_2_ was selected as an additive to the Sm_0.2_Ce_0.8_O_2-δ_ (SDC) electrolyte and its influence on the electrolyte properties were investigated. The tetrabutyl titanate hydrolysis product was introduced into the SDC samples as a source of TiO_2_. The lattice contraction of SDC was observed by XRD when the smaller ionic radius Ti^4+^ (0.605Å) were substituted for Ce^4+^ (0.97 Å). XRD analysis shows that the doping content of the TiO_2_ in SDC should be limited to 1 wt% to maintain the single-phase cubic fluorite structure of the SDC and avoid impurity phases. SEM characterizations suggest that the addition of TiO_2_ significantly promoted the grain growth and the sintering activity, especially when doping with 0.5 wt% of TiO_2_. The electrochemical measurements reveal that the addition of TiO_2_ had little effect on the conductivity of SDC samples, which was 0.0306 S cm^−1^ at 700°C. This study shows that 0.5 wt% TiO_2_ doping can effectively improve the sintering activity without reducing the SDC performance.

## 1 Introduction

As one of the most promising energy conversion technologies, solid oxide fuel cells (SOFCs) have been widely investigated and utilized in the past few decades owing to their low pollution emission and high conversion efficiency (Chen et al., 2020; Sun et al., 2021; Tahir et al., 2022). Many efforts have been made to advance the application of SOFCs in large-scale power plants and various transportation systems (Park et al., 2014; Curletti et al., 2015; Chen et al., 2018; Lee et al., 2018; Zhang et al., 2022). As an important component of SOFCs, the electrolyte acts as a gas barrier between the anode and the cathode, preventing the fuel and air from mixing. Meanwhile, the electrolyte offers a pathway for oxygen transportation due to the presence of oxygen vacancies in the lattice, which play a role in facilitating the ionic oxygen conductivity (Norberg et al., 2011; Mahato et al., 2015).

Among the many candidates, yttrium stabilized zirconia (YSZ) has a high oxygen ionic conductivity and chemical stability and is a commonly used electrolyte for SOFCs (Son et al., 2020). However, the utilization of the YSZ electrolyte requires a high operating temperature since its ionic conductivity drastically decreases below 800°C (Chen et al., 2002; Zheng et al., 2011; Zhao et al., 2013). Additionally, Bi_2_O_3_, which shows high ionic conductivity at intermediate temperatures, is also considered to replace YSZ (Punn et al., 2007; Li et al., 2008; Tan et al., 2012; Zagaynov et al., 2017). Another promising electrolyte family is Sr and Mg-doped lanthanum gallium (La_0.8_Sr_0.2_Ga_0.8_Mg_0.2_O_3-δ_, LSGM), which has a perovskite structure, high ion conductivity and negligible electronic conductivity at 600°C (Gao et al., 2020). In addition, it is chemically stable within a wide range of oxygen partial pressures (Garcia-Garcia et al., 2020). However, when the sintering temperature is higher than 1,250°C, the commonly used NiO-containing cermet anode tends to react with the LSGM electrolyte, generating the second phase with high resistance at the anode**/**electrolyte interface, which leads to the degradation of cell performance (Joo et al., 2011).

Therefore, developing alternative electrolytes which show high ionic conductivity at intermediate temperatures is urgent. In recent years, studies have shown that the ionic conductivity of doped CeO_2_ is nearly one order of magnitude higher than that of YSZ at 500**∼**800°C (Raghvendra and Singh, 2017). Moreover, there is no phase transition for Sm_0.2_Ce_0.8_O_2-δ_ (SDC) from room temperature to high temperatures. The addition of the trivalent rare-earth ions Y^3+^, Gd^3+^ or Sm^3+^ into the CeO_2_ lattice can form a cubic fluorite structure and generate a large number of oxygen vacancies, which further improves the ionic conductivity and mobility (Zheng et al., 2011; Anjaneya et al., 2014). In particular, in Sm-doped CeO_2_ materials, the radius of Sm^3+^ (1.04 Å) matches the radius of Ce^4+^ (1.04 Å), and the binding enthalpy between Sm^3+^ and the oxygen vacancies in the CeO_2_ lattice is the lowest, which is most favorable for oxygen ion transport. Therefore, SDC has a high ionic conductivity at low and medium temperatures.

Despite the excellent ionic conductivity of SDC, there are still two main issues limiting its application as electrolytes for SOFCs, i.e., the poor sintering activity and the increase of electrical conductance at low oxygen partial pressures. Due to the space charge effect, Sm doping has a significant inhibitory effect on the grain growth of CeO_2_, which is unfavorable to SDC sintering.

$${Sm}_{2}O_{3}\to 2{Sm}_{Ce}^{\prime }+3O_{O}^{\times }+V_{O}^{∙∙}$$

A certain number of oxygen vacancies ( $V_{O}^{∙∙}$ ) are generated in SDC, and these vacancies tend to accumulate at the grain boundary, making the accumulation region positively charged. The negatively charged dopant is attracted to the grain boundary region by Coulomb gravity, forming a dopant-rich layer in the grain boundary region, thus forming a doping gradient from the bulk phase to the grain boundary. This doping gradient is not conducive to grain boundary migration, and inhibits grain growth. In order to reduce the sintering temperature of SDC, additives can be added to reduce the sintering temperature of the electrolyte. In this paper, we propose to use TiO_2_ as a sintering additive to inhibit the migration of oxygen vacancies in SDC to the grain boundary. Grain boundary mobility is influenced by dopant-defect interaction which is charge and size dependent. The ionic radius of Ti^4+^ (0.605 Å) does not match the radius of Ce^4+^ (1.04 Å), which may significantly increase the tendency to enhance grain boundary mobility for CeO_2_, due to the large distortion of the surrounding lattice that apparently facilitates defect migration. The introduction of Ti^4+^ breaks the effect of space charge effect introduced by Sm-doped CeO_2_, which not only facilitates the growth of SDC grains, but also the bulk phase conduction of oxygen ions in SDC.

The addition of 0.1 mol% TiO_2_ to the CeO_2_ matrix has been found to enhance the grain boundary mobility of cerium oxide (Chen and Chen, 1996). However, additives that act as sintering aids in the SDC electrolyte may cause problems, such as the appearance of impurity phases and the reduction of ionic conductivity. In this work, TiO_2_ were selected as additives to improve the sintering activity of SDC. The influence of TiO_2_ addition on the phase stability, microstructure, conductivity and sintering behavior of SDC was investigated.

## 2 Experimental

Commercial SDC powder (Samarium Doped Ceria (20% Sm)-Tape Cast Grade Powder, Fuel Cell Materials, United States) with a surface area of 5.8 m^2^ g^−1^ was used in this study. TiO_2_ is obtained by hydrolysis and calcination of tetrabutyl titanate solution (C_16_H_36_O_4_Ti, 99.0%; Tianjin Kermel, China). SDC powders were mixed with tetrabutyl titanate using ethanol as solvent, and 1 wt% of PVB was added into the starting solution. The raw material powder was obtained by stirring and drying the solution in a hot plate. The SDC powder of mixed TiO_2_ (0–1.5 wt% of TiO_2_ to SDC denoted as SDC-xT; x = 0, 0.1, 0.2, 0.5, 1 and 1.5) was pressed with a die of diameter 13 mm at 5 MPa. The initial diameter (Ø_0_) of the sample is 13 mm. The pellets were subsequently sintered in air for 5 h at 1,400°C, 1,300°C and 1,200°C, respectively. The diameter of the calcined disc was measured with vernier calipers as Ø_1_. The shrinkage of the calcined pellets was calculated as Ø_1_/ Ø_0_. The density of the samples were obtained by Archimedes’ drainage method.

The X-ray diffraction (XRD) data of the SDC-XT pellets were obtained using a Bruker D8 advance (Germany, diffractometer with Cu-Kα radiation, scan test parameters: 20–80°, scanning speed: 5° min^−1^). The valence state of cerium was investigated by X-ray photoelectron spectroscopy (XPS, Escalab 250Xi). All the spectra were calibrated with the binding energy of carbon (1s) as the baseline (284.6 eV). The curve fitting of the XPS spectra was achieved by the XPS speak4.1 software.

For electrochemical characterizations, Pt slurry was coated on both sides of the SDC-xT pellets and fired at 800°C for 1 h. Electrochemical impedance spectroscopy (EIS) was performed on symmetric cells in static air, in a temperature range of 550–800°C, using the electrochemical workstation (Zennium) in the frequency range from 0.01 Hz to 1 MHz. The ionic conductivity was calculated using the measured resistance according to the following equation:

σ=L/(R∗S) (1)

where *σ* is the ionic conductivity (S cm^−1^), *L* is the pellet thickness (cm), and *S* is the surface area of the electrode (cm^2^). Each measurement was taken three times and the results were averaged for further comparison.

The microstructures of the electrolyte samples were characterized using scanning electron microscopy (SEM, HT7700 Exalens). The fractured cross-section and surface of the disc samples were sputter-coated with gold for SEM observations. The average grain size was estimated by the Nano Measurer software.

## 3 Results and discussion

**FIGURE 1:**
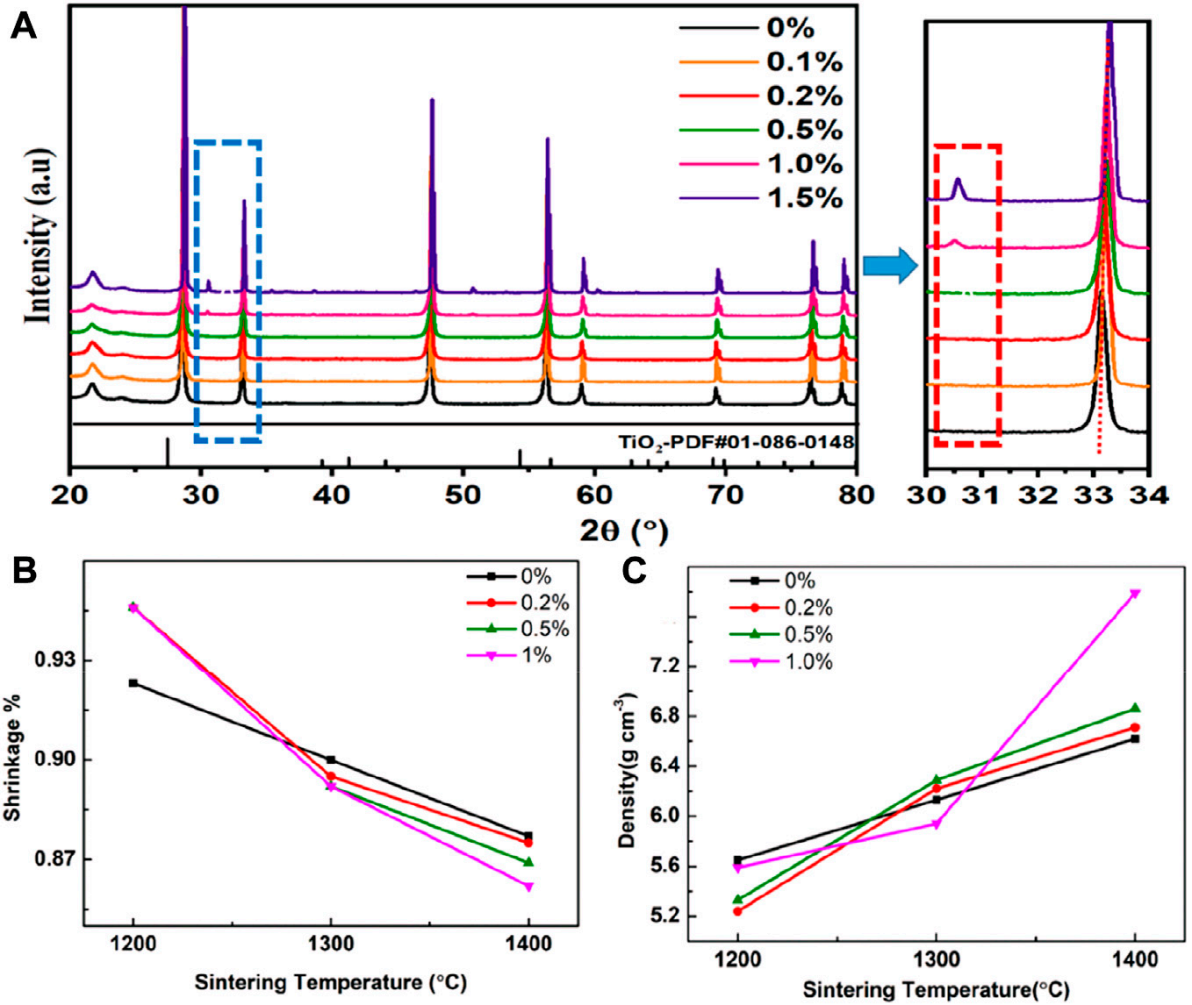
**(A)** The XRD patterns of SDC-xT with different TiO_2_ contents (x = 0, 0.2, 0.5, 1.0 and 1.5). **(B)** The shrinkage of SDC-xT (x = 0, 0.2, 0.5 and 1). **(C)** The density of SDC-xT (x = 0, 0.2, 0.5 and 1) sintered at different temperatures.

Figure 1A shows the XRD spectra of the SDC-xT (x = 0, 0.2, 0.5, 1 and 1.5) samples sintered at 1,400°C for 5 h. The SDC-xT (x = 0, 0.2 and 0.5) samples were found to have single phase with a cubic-fluorite structure. When the TiO_2_ content is below 1%, TiO_2_ is better dispersed in SDC and both form a good solid solution. When the TiO_2_ content exceeded 1 wt%, impurity phases were observed and the intensities of the impurity phases increased with the TiO_2_ content. Rutile TiO_2_ (JCPDS^#^-010860148) has a characteristic peak at 27.4°, and anatase TiO_2_ has a characteristic peak at 25.3°. The above characteristic peaks cannot coincide with the impurity peak in Figure 1A, which proves that TiO_2_ and SDC have chemically reacted to produce a new substance. When the TiO_2_ content exceeded 1%, the reaction between TiO_2_ and SDC produced CeTi_2_O_6_ (Otsuka-Yao-Matsuo et al., 2004), and this impurity phase could also be observed in the SEM spectrum of the sample.

With the increase of TiO_2_ content, the characteristic peaks of the XRD were shifted to higher angles, which proved that the samples exhibited obvious lattice shrinkage. The cell volume of each SDC sample was evaluated by fitting the XRD patterns using the Jade software. Table 1 summarized the impact of TiO_2_ content on the cell volume. The cell volume decreases with TiO_2_ content, except for SDC0.5T, which shows a slight increase in cell volume. The radius of the ions decreases in the following order: Ce^3+^ (1.283 Å) > Sm^3+^ (1.219 Å) > Ce^4+^ (0.97 Å) > Ti^4+^ (0.74 Å)/Ti^3+^ (0.67 Å) (Shannon, 1976; Mao et al., 2010; Li et al., 2013). Thus, the partial substitution of Ce^4+^ with Ti^4+^/Ti^3+^ leads to a decrease in cell volume, exhibits significant lattice shrinkage. However, when the TiO_2_ content is 0.5 wt%, the cell volume increases. According to the XPS spectra of Ce 3d and O 1s (Figure 3), when doping with 0.5 wt% TiO_2_, the incorporation of TiO_2_ promotes the reduction of Ce^4+^ to Ce^3+^, corresponding to an increase in cell volume (Mandal et al., 2016). Meanwhile, the increase in adsorbed oxygen suggested by the O 1s spectrum also accounts for the increase in cell volume.

**TABLE 1:** Cell volumes derived from the XRD patterns of samples with different TiO_2_ content.

	0 wt%	0.2 wt%	0.5 wt%	1.0 wt%	1.5 wt%
Cell volume (Å)	158.97	158.77	158.84	158.33	158.00

The density of the samples were obtained by Archimedes’ drainage method. The shrinkage (Figure 1B) and densification (Figure 1C) of the SDC were investigated as a function of TiO_2_ content and sintering temperature. Figure 1B shows the shrinkage of the SDC-xT with sintering temperature. The shrinkage was significantly high for samples sintered at higher temperatures compared to lower temperatures. Therefore, the optimum sintering temperature for the SDC-xT powders was selected to be 1,400°C. The density of all SDC-xT samples increase with the increase in sintering temperature, as shown in Figure 1C. After sintering at 1,200°C, the undoped SDC-0T showed the highest density and the density of the doped SDC-xT decreased with increasing of TiO_2_ content. For the samples sintered at 1,400°C, the densities of all SDC-xT samples showed a large increase, with SDC-1T showing a drastic increase.

**FIGURE 2:**
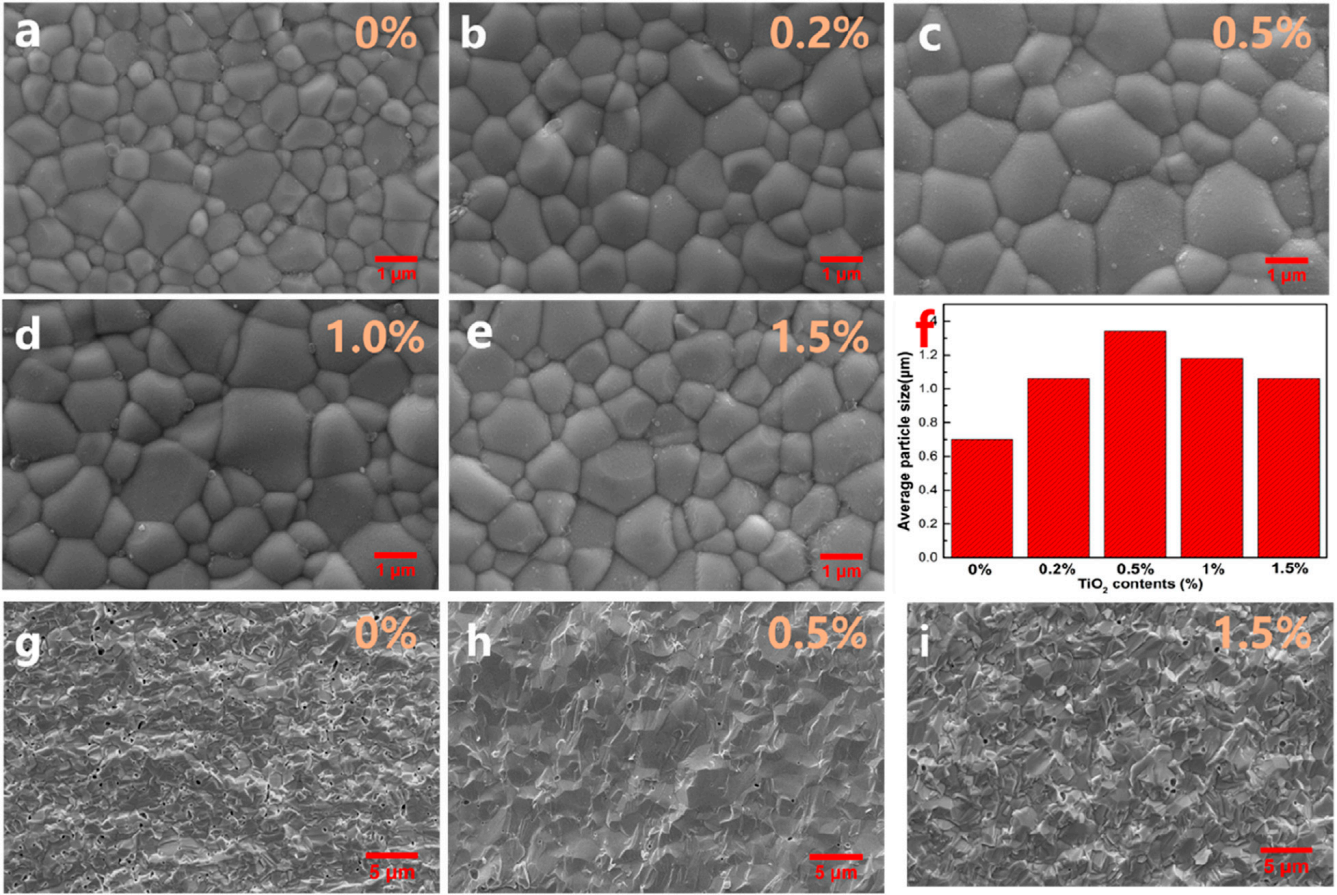
**(A–E)** The surface SEM images of SDC-xT (x = 0, 0.2, 0.5, 1 and 1.5) sintered at 1,400°C. **(F)** The averaged particle sizes for SDC-xT (x = 0, 0.2, 0.5, 1 and 1.5). **(G–I)** The cross-sectional SEM images of SDC-xT (x = 0, 0.5 and 1.5).

Figures 2A–E show the surface SEM images of SDC-xT (x = 0, 0.2, 0.5 1 and 1.5). The grain size was obtained by multiplying the average linear intercept length of at least 200 grains by Nano Measurer software. The averaged particle sizes for SDC-xT were shown in Figure 2F. With the increase of TiO_2_ content, the size of grains shows a parabolic trend, the average particle size of the SDC-xT samples first increases and then decreases. The average particle size for SDC-0.5T reached 1.34 μm, which is nearly twice as large as that of undoped SDC. Figures 2G–I provide the cross-sectional SEM images of SDC-xT (x = 0, 0.5, and 1.5). It is clearly observed from the fracture that the addition of TiO_2_ can improve the sintering density and reduce the porosity of the samples, and TiO_2_ addition is beneficial to the densification of SDC electrolytes. The sample had the smallest porosity and the densest when the TiO_2_ content in the sample was 0.5%. The SDC-0.5T had the largest grain size and the smallest porosity. Severely undersized dopants TiO_2_ at lower doping have a tendency to enhance grain boundary mobility, probably due to the large distortion of the surrounding lattice that apparently facilitates defect migration. However, TiO_2_ at higher doping have a tendency to suppress grain boundary mobility for a strong solute drag effect (Chen and Chen, 1996).

Since the activation energy for oxygen ions conductivity increases as the grain size decreases, a large grain size offers an easy pathway for oxygen transport in the electrolyte. Meanwhile, large grain size can also reduce the alternating current (AC) impedance of internal grains (Pei et al., 2017), facilitating the conduction of oxygen ions in the electrolyte. The surface morphology of the SDC-xT suggests that doping SDC with 0.5 wt% TiO_2_ is the best composition to achieve a dense electrolyte, and 1,400°C is the appropriate sintering temperature.

**FIGURE 3:**
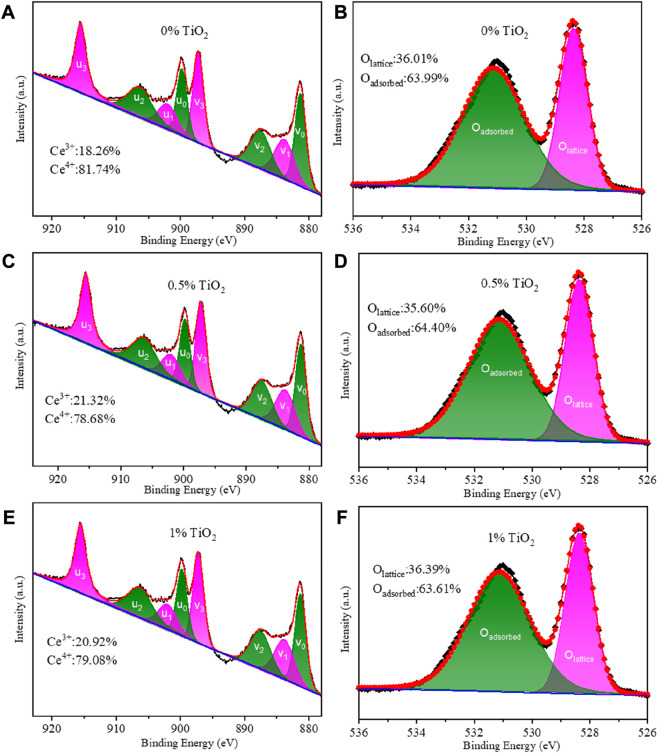
**(A,C,E)** XPS Ce 3d emission spectra of SDC-xT (x= 0, 0.5 and 1); **(B,D,F)** XPS O1s emission spectra of SDC-xT (x= 0, 0.5 and 1).

XPS was utilized to investigate the influence of TiO_2_ content on the oxidation state of Ce. Figures 3A,C,E show the XPS spectra of Ce 3d; the peaks are denoted as u and v, corresponding to Ce 3d_3/2_ and Ce 3d_5/2_, respectively. The sub-bands denoted as u_3_, v_3_, u_1_ and v_1_ are assigned to the characteristic peaks of Ce^4+^, while the sub-bands labeled u_2_, v_2_, u_0_ and v_0_ correspond to the characteristic peak of Ce^3+^ (Heckert et al., 2008; Gupta et al., 2009; Paparazzo, 2011). The Sm^3+^-doped CeO_2_ is a mixed ionic and electronic conductor, which exhibits the highest oxygen ionic conductivity at an optimal dopant concentration due to the minimal enthalpy of association between the Ce cations and oxygen vacancies in the fluorite lattice (Chen et al., 2009). The Ce^4+^/ Ce^3+^ ratios in SDC-xT with different TiO_2_ content are given in Figures 3A,C,E. As shown in Figure 3A without TiO_2_, the Ce^3+^ content in SDC was 18.26%. When the TiO_2_ content is 0.5%, the Ce^3+^ content in SDC-0.5T increased to 21.32% (Figure 3C). However, when the TiO_2_ content was 1%, the ratio of Ce^4+^/Ce^3+^ decreases, the content of Ce^3+^ decreased to 20.92% (Figure 3E), which can be attributed to an excess of TiO_2_ that does not enter the CeO_2_ lattice and forms impurity phases, such as CeTi_2_O_6_^25^.

Figures 3D–F show the XPS spectra of O 1s. The sub-band with a binding energy of 528.5 eV represents the characteristic peak of lattice oxygen, and the sub-band with a binding energy of 531 eV corresponds to the characteristic peak of adsorbed oxygen (Kang et al., 2019). Figure 3 gives the ratio of lattice oxygen/adsorbed oxygen for SDC-xT samples with different TiO_2_ content. The lattice oxygen content of SDC-0.5T is 35.60% and the adsorbed oxygen content is 64.40%. It can be seen from Figure 3 that when the content of Ce^3+^ increases, the content of lattice oxygen decreases and the content of adsorbed oxygen on the oxide surface increases. SDC-0.5T has the highest surface adsorbed oxygen content. This may be due to the distortion of the lattice part caused by the mismatch of ionic radii when Ti^4+^ occupies the lattice site of Ce^4+^. The radius of Ce^3+^ (1.14 Å) is larger than that of Ce^4+^ (0.97 Å), and in order to attenuate the degree of distortion, part of Ce^4+^ is reduced to Ce^3+^, which results in a decrease of the lattice oxygen content and an increase of the surface oxygen content.

**FIGURE 4:**
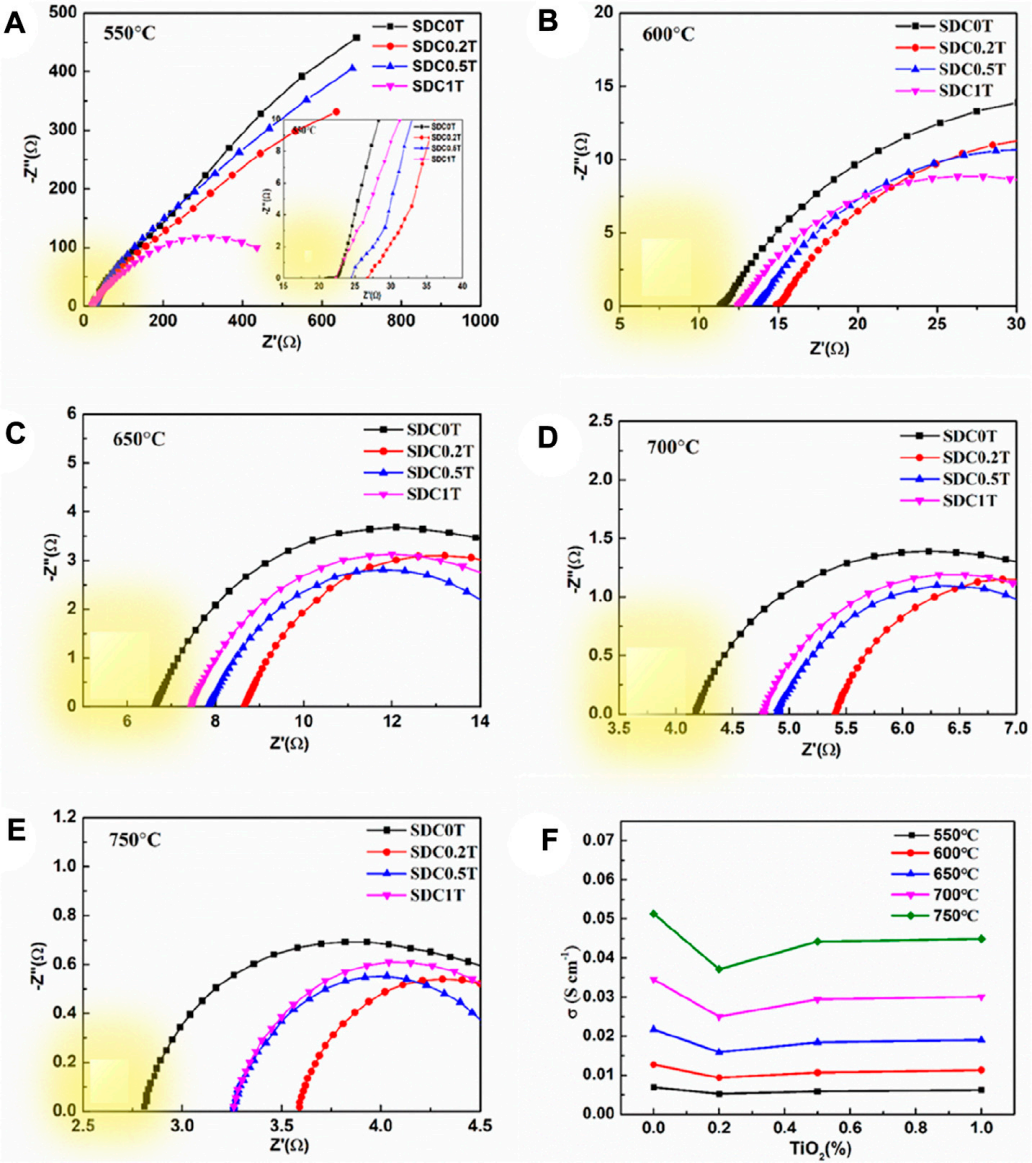
**(A–E)** Nyquist diagram of the impedance spectra for SDC-xT obtained at 1,400°C. **(F)** The electrical conductivity of the SDC-xT from 550°C to 750°C.

The ohmic resistance of SDC-xT (x = 0, 0.2, 0.5, and 1) sintered at 1,400°C was obtained using the four-probe method in the temperature range of 600–750°C, as shown in Figures 4A–E. In the Nyquist plot, the intersection of the impedance data measured at high frequencies with the x-axis (the real part of the impedance) is the ohmic resistance indicated by the yellow color block in the figure. The ohmic resistance value decreases with increasing test temperature, indicating that the conductivity of the electrolyte is proportional to temperature. The electrical conductivity of the SDC-xT can be calculated using Eq. 1, and the result are shown in Figure 4F. Table 2 gives the electrical conductivity of SDC-xT as a function of TiO_2_ content and sintering temperatures. mThe temperature firstly has a very significant effect on the conductivity, and secondly the content of TiO_2_ also has an effect on the conductivity. As an example, the conductivity of the sample at 650°C was 0.0217 s cm^−1^ without the addition of TiO_2_, and the conductivity of the sample decreased to 0.0169 s cm^−1^ with 0.2 wt% TiO_2_ was added. When the TiO_2_ content was increased to 0.5 wt% and 1.0 wt%, the conductivity of SDC-0.5T was similar to that of SDC-1.0T.

**TABLE 2:** Ideal electrical conductivity of SDC-xT between 600–750°C.

σ (S/cm)	600°C	650°C	700°C	750°C
0 wt.%TiO_2_	0.0127	0.0217	0.0345	0.0513
0.2 wt.%TiO_2_	0.0097	0.0169	0.0269	0.0407
0.5 wt.%TiO_2_	0.0118	0.0197	0.0306	0.0450
1.0 wt.%TiO_2_	0.0118	0.0198	0.0309	0.0454

The density and shrinkage characterizations of the SDC-xT samples show that the samples sintered at 1,400 °C demonstrate the best sintering activity and largest grain size, which is beneficial for oxygen transport. As expected, the conductivity of undoped SDC sintered at 1,400°C was higher than that of undoped SDC sintered at lower temperatures. However, TiO_2_ addition led to slight reduction in the conductivity of SDC-xT, which can be attributed to the poor conductivity of TiO_2_ (< 10^–10^ S cm^−1^) (Miyazaki, 2008; Mazúr et al., 2012). Upon TiO_2_ addition, Ti^4+^ enters the SDC lattice, partially replacing Ce^4+^, resulting in a decrease in oxygen vacancy concentration of SDC (Miyazaki, 2008); consequently, decreasing the conductivity of SDC. In addition, according to the SEM results, the densification of SDC increases upon TiO_2_-doping when x = 0.2 and 0.5. While the conductivity of the SDC-xT samples slightly decreased, it still qualifies for electrolyte applications in SOFCs (Li et al., 2006; Bu et al., 2013). Subsequently, SDC-0.5T sintered at 1,400°C, with large particle size and good electrical conductivity, is a suitable electrolyte candidate.

**FIGURE 5:**
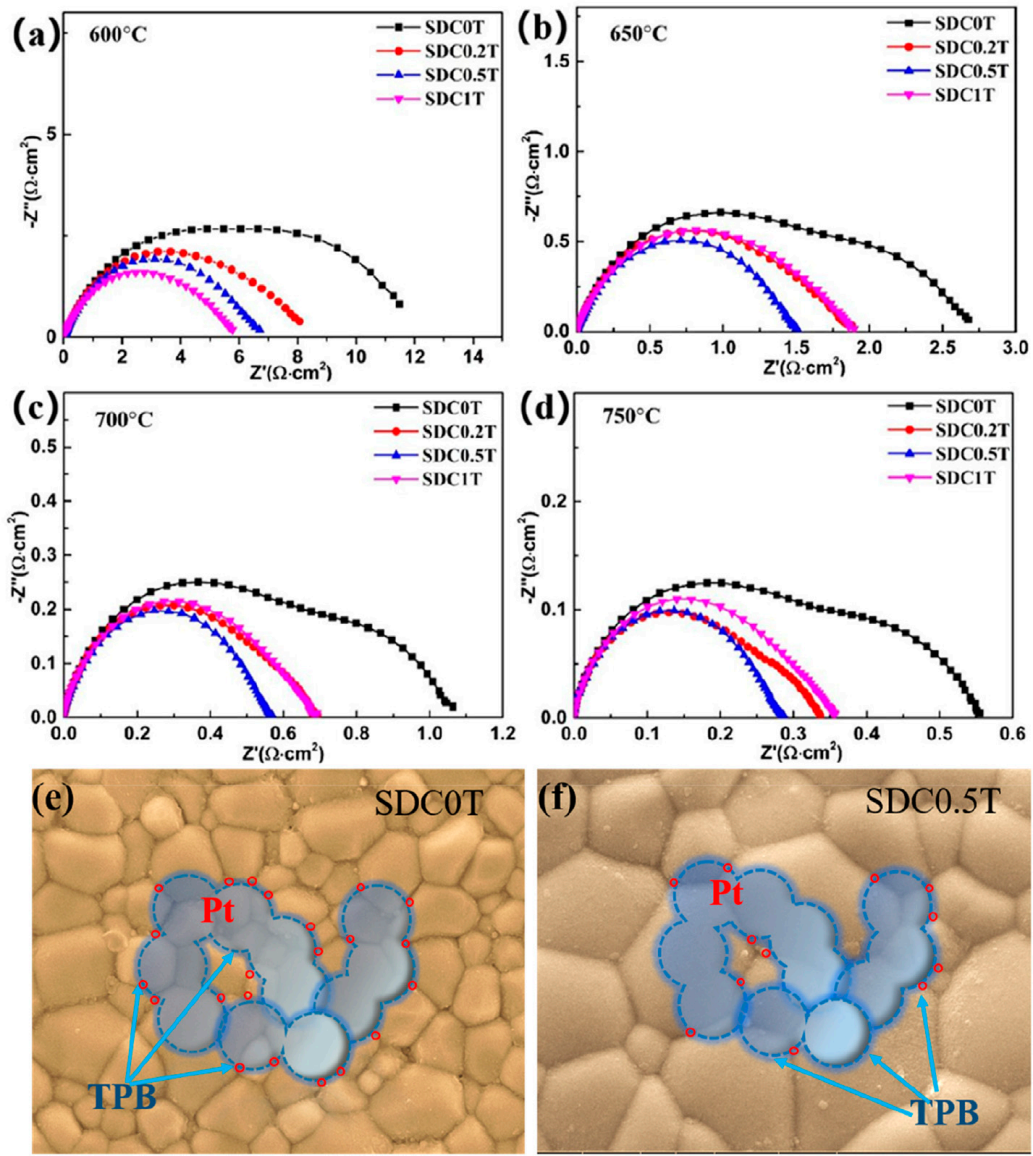
**(A–D)** Nyquist plots of the impedance spectra of the Pt electrodes. **(E–F)** Schematic diagram of the three-phase boundary of the Pt electrode reacting with oxygen at SDC-0T and SDC-0.5T electrolyte surface.

Using Pt as the electrodes for symmetrical cells, the EIS results at different temperatures are shown in Figures 5A–D. Generally, the oxygen reduction reaction at the cathode includes the surface path and volume path (Fleig, 2003). However, for the pure electronic conductor Pt (Barbucci et al., 2002), the oxygen reduction reaction can only be carried out at the three-phase boundary (TPB) interface between the electrode, electrolyte and oxygen. As can be seen from the schematic diagram Figures 5E,F, the blue dashed line is the TPB interface. Hence, for Pt electrodes, only the TPB is the active reaction region (Co and Birss, 2006).

The reaction of oxygen at the electrode mainly includes several processes such as gas-phase diffusion, dissociation-adsorption, surface diffusion, and charge transfer.

$$O_{2}⇆O_{2,ads}$$ (2)

$$O_{2,ads}⇆2O_{ads}$$ (3)

$$O_{ads}+2e^{-}+V_{O}^{..}⇆V_{o}^{x}$$ (4)

The high frequency region of the EIS mainly corresponds to the charge transfer process, which is a fast reaction process. The low frequency region corresponds to the gas-phase diffusion process and dissociation-adsorption process. As shown in Figure 5, the polarization resistance of Pt at 650–750°C in the TiO_2_-doped electrolyte is smaller than that of the undoped SDC-0T. The polarization resistance of SDC-0.5T is the smallest, and the polarization resistance of SDC-0.2T is similar to that of SDC1T. At 700°C, the EIS of SDC0T clearly shows two arcs in the high-frequency region and low-frequency region, and the arc in the high-frequency region is larger than that in the low-frequency region. With the addition of TiO_2_, both arcs are reduced. The reduction of the arc in the high frequency is not significant, but the arc in the low frequency is significantly reduced. The experimental results indicate that the change in grain size of the electrolyte leads to a change in the rate control process of the oxygen reduction reaction. The charge transfer process corresponding to the high-frequency region of the impedance spectrum is less influenced by the grain size, while the adsorption-diffusion of oxygen in the corresponding low-frequency region are strongly influenced by the grain size.

Although the length of TPB interface of Pt on the surface of SDC-0T and SDC-0.5T electrolytes is the same as shown in Figures 5E,F, the Pt electrode forms a larger contact point with the grain boundary on the surface of small-sized grains (SDC-0T) than on the surface of large-sized grains (SDC-0.5T) due to the difference in grain size. The contact points are shown as red circles in the figure.

There are two paths of oxygen ions transport, one way is oxygen ions through the grain and the other way is oxygen ions through the grain boundary. The impedance spectrum shows that the polarization resistance of the electrode is gradually becoming smaller as the electrolyte grain size becomes larger, especially the impedance in the low frequency region decreases ---significantly. This result indicates that the grain boundaries in the SDC electrolyte are not favorable for the oxygen adsorption-desorption reaction, and the oxygen ions are more favorable for conduction through the grain body phase. The large grain size can effectively reduce the interface between TPB and grain, which reduces the resistance to grain boundary conduction and enhances the bulk phase conduction of oxygen ions, thus facilitating steps (2)–(3). Tian et al. (Tian and Chan, 2000) found that an increase in the sintering temperature of SDC leads to a decrease in the grain boundary region when the grain size grows, and increases the impurities within the grain boundaries as well as the Sm_Ce_´ concentration, which brings about a decrease in the grain boundary conductivity. The results also demonstrate that the growth of SDC grain size leads to a reduction in the grain boundary region, which brings about a decrease in grain boundary conductivity.

## Conclusion

The electrolyte performance of SDC with the addition of TiO_2_ at various ratios was first explored. After sintering at different temperatures, it was found that the SDC had the best sintering activity at 1,400°C. In particular, when the doping ratio is 0.5 wt%, TiO_2_ not only promoted the increase in the electrolyte grain size but also had a minimal effect on the conductivity of the SDC. The bigger SDC grain size leads to a reduction in the grain boundary region and brings about a decrease in grain boundary conductivity.

The conductivity of SDC-0.5T reached 0.0306 S cm^−1^ at 700°C. This proves that TiO_2_ is an excellent sintering aid, and 0.5 wt% is considered to be the best dopant concentration. When the doping rate was 0.5 wt%, the addition of TiO_2_ improved the sintering activity of SDC, leading to an increased grain size, which facilitated the oxygen transport in the electrolyte.

## Data Availability

The original contributions presented in the study are included in the article/supplementary materials, further inquiries can be directed to the corresponding author.
